# Supplementary material for: Complex‐centric proteome profiling by SEC‐SWATH‐MS
Source: Mol Syst Biol. 2019 Jan 14;15(1):e8438. doi: 10.15252/msb.20188438 (PMC6346213; doi:10.15252/msb.20188438)
Supplement: Supplementary file 8 — Dataset EV7 [file MSB-15-e8438-s008.zip › feature_plots_string/Q92925.pdf]

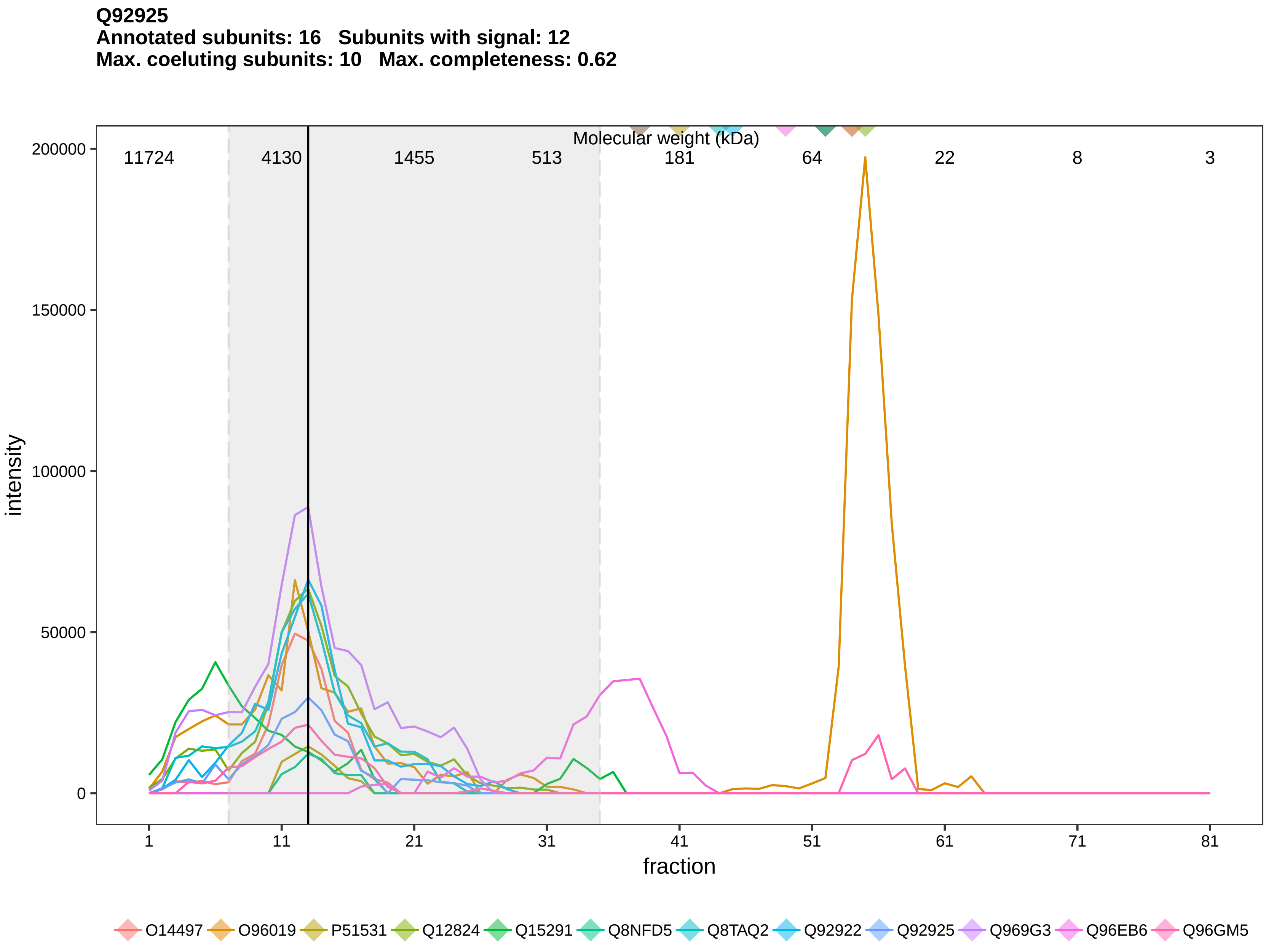

Q92925
Annotated subunits: 16   Subunits with signal: 12
Max. coeluting subunits: 10   Max. completeness: 0.62
Molecular weight (kDa)
11724
4130
1455
513
181
64
22
8
3
intensity
200000
150000
100000
50000
0
1
11
21
31
41
51
61
71
81
fraction
O14497
O96019
P51531
Q12824
Q15291
Q8NFD5
Q8TAQ2
Q92922
Q92925
Q969G3
Q96EB6
Q96GM5
